# Computational Assessment of CKP-25 Binding at Receptor-Recognition Interfaces Across Diverse Human Coronaviruses

**DOI:** 10.3390/ph19101664

**Published:** 2026-10-21

**Authors:** Angelos-Michail Papadopoulos, Spyros Petrakis, Federico Alvarez, Petros Daras

**Affiliations:** 1 Information Technologies Institute, Centre for Research and Technology Hellas (CERTH), 6th km Charilaou–Thermi Road, Thermi, 57001 Thessaloniki, Greece; daras@iti.gr; 2 Grupo de Aplicación de Telecomunicaciones Visuales, Information Processing and Telecommunications Center, Universidad Politécnica de Madrid, 28040 Madrid, Spain; federico.alvarez@upm.es; 3 Institute of Applied Biosciences, Centre for Research and Technology Hellas (CERTH), 6th km Charilaou–Thermi Road, Thermi, 57001 Thessaloniki, Greece

**Keywords:** coronavirus, CKP-25, molecular docking, molecular dynamics, receptor-binding interface, MM-GBSA, in silico binding assessment

## Abstract

**Background/Objectives:** The continued emergence of novel coronaviruses motivates the search for small molecules that bind conserved features of the spike receptor-recognition interface across diverse species. CKP-25 was recently reported as a small-molecule inhibitor of the SARS-CoV-2 spike receptor-binding domain. This study aimed to assess computationally whether CKP-25 can maintain stable binding at receptor-recognition interfaces across phylogenetically diverse human coronaviruses. **Methods:** CKP-25 was docked to the spike receptor-binding regions of Middle East respiratory syndrome coronavirus (MERS-CoV), severe acute respiratory syndrome coronavirus 1 (SARS-CoV-1), and human coronaviruses NL63, 229E, and HKU1 (HCoV-NL63, HCoV-229E, and HCoV-HKU1). SARS-CoV-2 (PDB 6M0J), for which CKP-25 has documented antiviral activity, was processed identically and included as an experimentally validated reference system. Each selected complex was subjected to three independent 100 ns all-atom molecular dynamics (MD) simulations initialized with different random velocities. Binding stability was evaluated using protein and ligand root-mean-square deviation (RMSD), residue flexibility, radius of gyration, protein–ligand contact persistence, polar interactions, and Molecular Mechanics–Generalized Born Surface Area (MM-GBSA) calculations. **Results:** CKP-25 was retained at the targeted receptor-recognition interface in all three replicates for four of the five test targets—MERS-CoV, HCoV-NL63, SARS-CoV-1, and HCoV-HKU1—each of which reproducibly engaged the same aromatic and hydrophobic anchor residues in every independent run. HCoV-229E was the sole exception, the ligand relocating from its docked site in two of three replicates. Ligand RMSD plateaus were generally low, mostly below 2.0 Å. Contact analyses indicated a shared dependence on shape-complementary van der Waals packing involving hydrophobic and aromatic residues, including tyrosine- and tryptophan-rich anchoring environments. MM-GBSA estimates, averaged across replicates, ranged from −24.28 to −34.57 kcal/mol. No test target differed from the SARS-CoV-2 reference (−29.15±3.46 kcal/mol) by more than the replicate-to-replicate spread, with MERS-CoV and HCoV-NL63 giving the most favorable means. **Conclusions:** CKP-25 remained at the receptor-recognition interface of four of the five human coronaviruses tested, and it did so in all three replicates. In the SARS-CoV-2 control, the same pipeline independently reproduced the experimentally established binding mode, which supports the reliability of these predictions. HCoV-229E was the exception: the ligand left the docked site in two of the three replicates, and no contact was reproducible across all three repetitions. Experimental binding measurements and cell-based entry assays are required to test these predictions.

## 1. Introduction

The recurrent emergence of highly pathogenic human coronaviruses (HCoVs) over the past two decades, most notably Severe Acute Respiratory Syndrome Coronavirus (SARS-CoV-1), Middle East Respiratory Syndrome Coronavirus (MERS-CoV), and SARS-CoV-2, has underscored the pandemic potential of this viral family [1,2]. Alongside these highly virulent strains, endemic coronaviruses such as HCoV-NL63, HCoV-229E, and HCoV-HKU1 persistently circulate within the human population, contributing significantly to seasonal respiratory diseases [3,4]. The continuous evolution and cross-species transmission of coronaviruses motivate the search for small molecules that engage structurally conserved features shared across these viruses.

Viral entry into the host cell is a critical bottleneck in the coronavirus life cycle and represents a primary target for drug discovery [5]. This process is mediated by the viral Spike (S) glycoprotein, a massive homotrimeric fusion protein. While the specific host receptors vary among strains, for instance, SARS-CoV-1 and HCoV-NL63 utilize Angiotensin-Converting Enzyme 2 (ACE2), MERS-CoV binds Dipeptidyl Peptidase 4 (DPP4), and HCoV-229E relies on Aminopeptidase N (APN), the structural mechanism of receptor engagement is highly conserved [6,7,8]. Consequently, small molecules that bind at the receptor-binding interfaces of diverse spike proteins have been proposed as a structurally conserved point of intervention that may be less susceptible to escape mutations [9,10].

In this study, we computationally assess the binding of CKP-25 (C_19_H_16_N_2_O), a small molecule with reported activity against SARS-CoV-2 [11], at the receptor-recognition interfaces of other human coronaviruses. CKP-25 was previously shown to protect human cells from SARS-CoV-2 infection, significantly suppressing viral titers in vitro. That reported activity is attributed to its stable binding at the interface between the SARS-CoV-2 spike Receptor-Binding Domain (RBD) and its ACE2 receptor, which is widely expressed on the cell surface of the human respiratory system [12]. We hypothesized that CKP-25 would remain associated with receptor-recognition interfaces across phylogenetically diverse human coronaviruses by exploiting shared hydrophobic and aromatic physicochemical features, despite differences in sequence and host-receptor usage.

**Figure 1:**
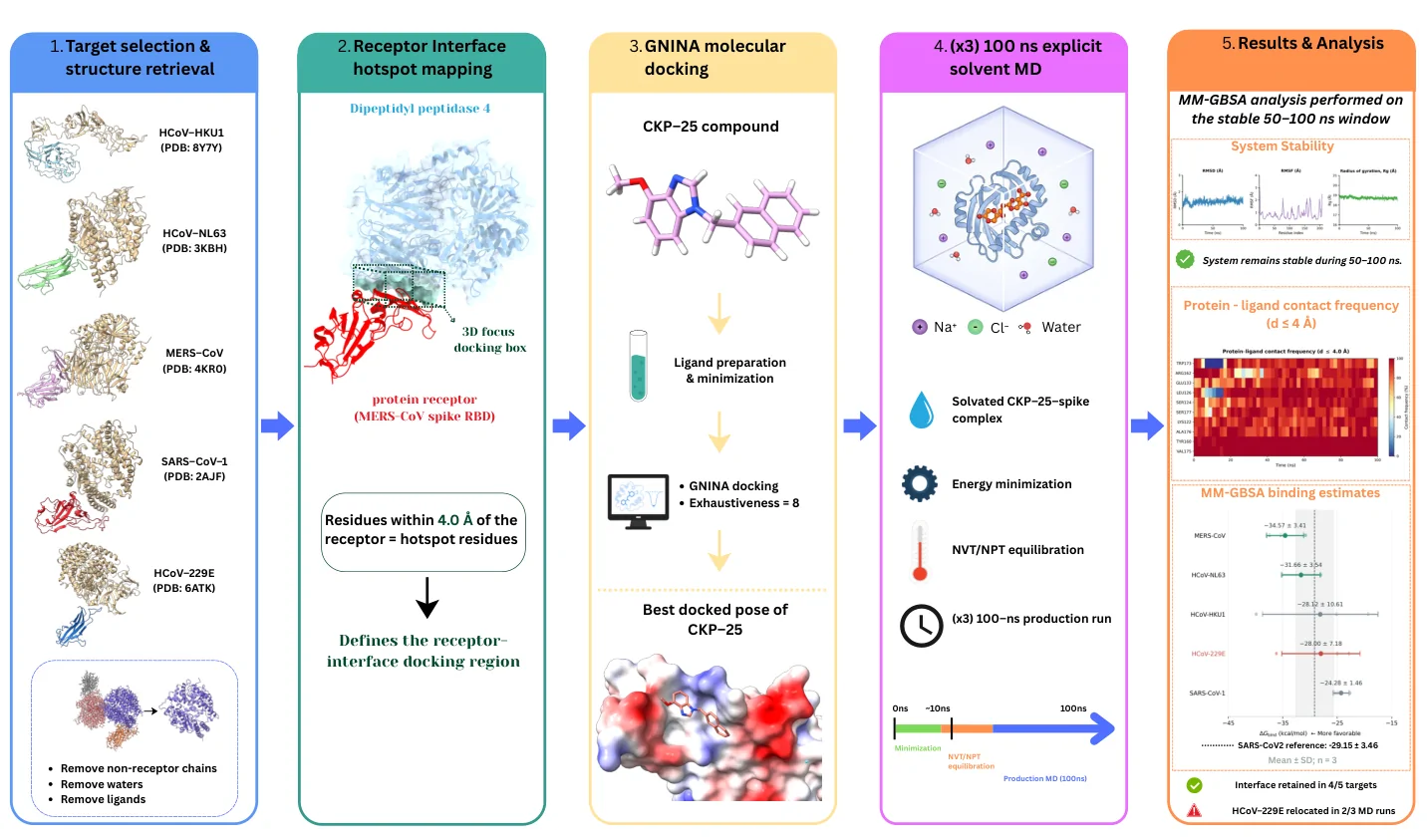
Computational workflow for evaluating CKP-25 binding across diverse human coronavirus spike interfaces. Experimentally determined spike receptor-binding structures from six human coronaviruses (PDB IDs: 8Y7Y, 3KBH, 4KR0, 6ATK, 2AJF, and 6M0J) were retrieved, and residues located within 4.0 Å of the corresponding host receptor or entry mediator were used to define target-specific receptor-interface docking regions. GNINA-guided molecular docking was then used to generate initial CKP-25 binding poses for three independent 100 ns explicit-solvent molecular dynamics (MD) simulations per target (eighteen trajectories in total). Structural stability metrics, including root-mean-square deviation (RMSD), root-mean-square fluctuation (RMSF), radius of gyration ($R_{g}$), and protein–ligand contact persistence, were evaluated over the complete production trajectories. Molecular Mechanics–Generalized Born Surface Area (MM-GBSA) calculations were performed using the equilibrated 50–100 ns window. All reported values are means across the three replicates, with uncertainties given as between-replicate standard deviations. SARS-CoV-2 (6M0J) serves as the experimentally validated reference against which the five test targets are compared. Panel 5 summarizes the outcome of this pipeline: representative stability traces (RMSD, RMSF and $R_{g}$) over the 50–100 ns window, the per-residue protein–ligand contact-frequency map at a 4.0 Å cutoff, and the replicate-averaged MM-GBSA binding estimates. In the latter, filled circles denote the mean across the three replicates and horizontal bars the between-replicate standard deviation, while the dashed line and shaded band mark the SARS-CoV-2 reference value (−29.15±3.46 kcal/mol). CKP-25 was retained at the receptor-recognition interface in all three replicates for four of the five test targets; HCoV-229E, shown in red, is the exception, with the ligand relocating to a different surface site in two of three replicates, so its energy does not describe binding at the receptor-recognition interface and is not comparable with the remaining four.

To assess the binding potential and structural stability of CKP-25, we employed an integrative computational pipeline combining molecular docking with extensive molecular dynamics (MD) simulations. While static docking provides initial binding poses, the highly dynamic nature of the spike protein, which undergoes large-scale conformational transitions between open (receptor-accessible) and closed (receptor-inaccessible) states of its receptor-binding domains (RBDs) [7,13], necessitates advanced physics-based simulations to validate the dynamic stability under the simulated conditions of the ligand–receptor complex [14]. Therefore, we subjected the CKP-25/spike complexes of five phylogenetically diverse coronaviruses (MERS-CoV, SARS-CoV-1, HCoV-NL63, HCoV-229E, and HCoV-HKU1) to three independent 100 ns all-atom MD simulations each, alongside the SARS-CoV-2 spike–ACE2 complex processed identically as an experimentally validated reference system [11]. By analyzing geometric stability metrics, tracking protein–ligand contact persistence, and calculating relative binding free-energy estimates via the Molecular Mechanics–Generalized Born Surface Area (MM-GBSA) method, we provide a comparative computational assessment of CKP-25 binding across these spike receptor-recognition interfaces. The overall computational workflow is summarized in Figure 1.

## 2. Results

### 2.1. Selected Human Coronavirus Spike Targets

Application of the structural selection criteria described in Section 4.1 yielded a panel of five experimentally resolved human coronavirus spike–host complexes: MERS-CoV (4KR0), SARS-CoV-1 (2AJF), HCoV-NL63 (3KBH), HCoV-229E (6ATK), and HCoV-HKU1 (8Y7Y), together with the SARS-CoV-2 spike–ACE2 complex (6M0J), which was carried through the identical workflow as an experimentally validated reference system. The panel included both alpha- and betacoronaviruses and represented distinct host receptors or entry mediators: ACE2, DPP4 and APN, together with TMPRSS2, which serves HCoV-HKU1 as a proteolytically independent binding partner and which we therefore designate an entry mediator rather than a classical receptor [15].

Native interface mapping identified a spatially defined receptor-recognition hotspot for each spike target. These target-specific hotspots were retained after removal of the host-partner coordinates and were used to direct focused docking of CKP-25. Thus, the final panel enabled comparative evaluation of CKP-25 at structurally and phylogenetically diverse coronavirus receptor-recognition interfaces.

### 2.2. Molecular Docking Affinities

Static molecular docking was performed to generate the initial binding poses of CKP-25 for the subsequent MD simulations. The GNINA-reported docking affinities indicated generally favorable interactions for CKP-25 across all targets (see Table 1). CNN pose scores for the selected poses spanned a narrow range (0.692–0.787), indicating that the starting structures submitted to MD were of comparable structural credibility across the panel. The reference system scored 0.739, so three of the five test targets began from poses scored at least as confidently as the pose that independently reproduces the published SARS-CoV-2 binding mode. The lowest score was obtained for HCoV-229E (0.692), the same system that also showed the smallest fraction of buried ligand surface.

**Figure 2:**
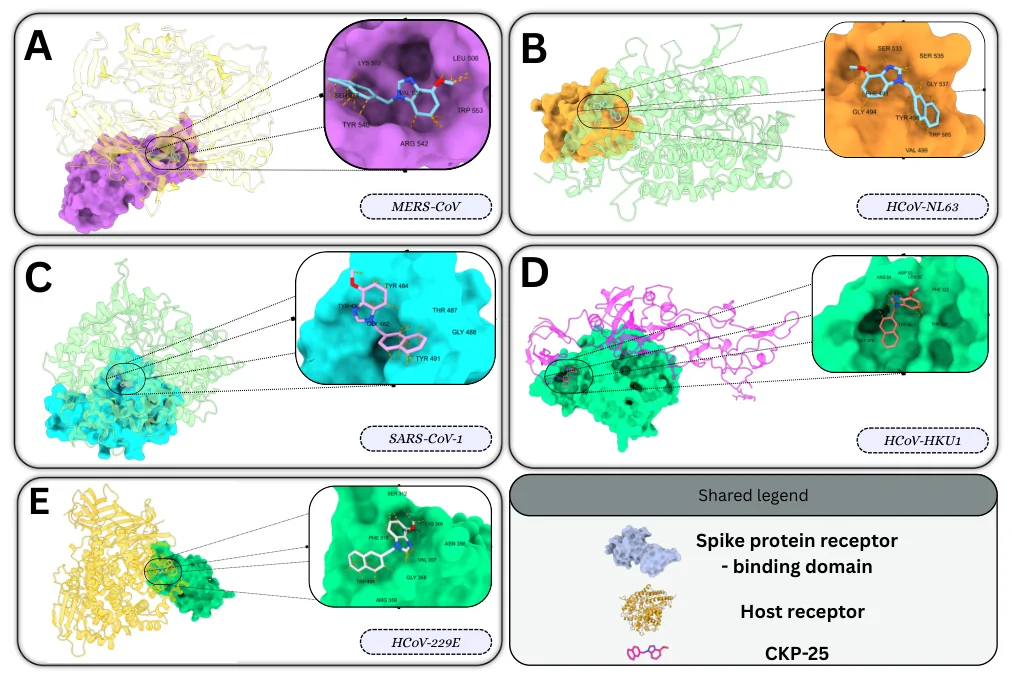
Starting molecular docking configurations of CKP-25 bound to the host-receptor interfaces of five target human coronavirus spike proteins. Panels show: (**A**) MERS-CoV (PDB 4KR0), (**B**) HCoV-NL63 (PDB 3KBH), (**C**) SARS-CoV-1 (PDB 2AJF), (**D**) HCoV-HKU1 (PDB 8Y7Y), and (**E**) HCoV-229E (PDB 6ATK). Each panel displays the full complex on the left with a zoomed-in inset on the right highlighting atomic-level contacts for key interface residues. The contacts labeled in panel (**E**) describe the docked pose rather than a persistent binding mode and should not be read as anchor residues in the sense used for the other four targets.

While all static docking affinity scores were encouraging (<−5.9 kcal/mol), it is crucial to note that they did not perfectly predict the final thermodynamic hierarchy observed in the dynamic MM-GBSA calculations (Section 2.6). This limitation is illustrated most directly by the reference system. SARS-CoV-2 is the only target in the panel for which CKP-25 has demonstrated antiviral activity, yet it ranks fifth of six by docking affinity (−6.03 kcal/mol), ahead of only HCoV-229E. Static docking score alone would therefore not have identified the one interface at which the compound is experimentally known to act, which underlines the need for the subsequent dynamic evaluation. For instance, HCoV-HKU1 achieved the best static docking affinity but demonstrated only moderate predicted binding after MD/MM-GBSA evaluation, whereas MERS-CoV and HCoV-NL63 exhibited more favorable predicted binding under dynamic simulation. This discrepancy highlights the importance of using 100 ns MD simulations to evaluate static docking poses, as the dynamic plasticity of the spike protein can significantly influence ligand stability and comparative binding rankings. Notably, this difference is not driven by major structural displacement of the ligand, as both MERS-CoV and HCoV-HKU1 show highly stable ligand RMSD plateaus (≤1.3 Å relative to the starting pose; see Section 2.3). Instead, the discrepancy is thermodynamic in nature: the HCoV-HKU1 interface offers fewer complementary hydrophobic packing partners ($\Delta E_{\mathrm{vdw}}=-31.74\pm 10.92$ kcal/mol) than the aromatic pocket of MERS-CoV ($\Delta E_{\mathrm{vdw}}=-38.89\pm 3.57$ kcal/mol), yielding a weaker mean binding free energy despite equivalent initial pose stability. The starting docked configurations, demonstrating the shape-complementary insertion of CKP-25 into the receptor-binding interfaces, are illustrated in Figure 2.

The docked pose obtained for the SARS-CoV-2 reference system provides an independent check on the docking protocol itself. The top-ranked pose (CNN pose score 0.739; Vina affinity −6.03 kcal/mol) placed CKP-25 in near-parallel π–π stacking with Tyr505, with a ring-centroid separation of 3.9 Å and an interplanar angle of $1^{\circ }$, together with contacts to Arg403, Tyr453, Ser494, Tyr495, Gly496, Gln498 and Asn501. This reproduces the binding mode previously reported for CKP-25 at this interface, in which π–π stacking on Tyr505 was identified as the interaction maintaining the compound in place [11]. Because that earlier characterization was obtained with the CHARMM36 force field, the agreement represents a force-field-independent recovery of the same pose rather than a reproduction of the same calculation.

### 2.3. System Equilibration and CKP-25 Binding Stability

**Figure 3:**
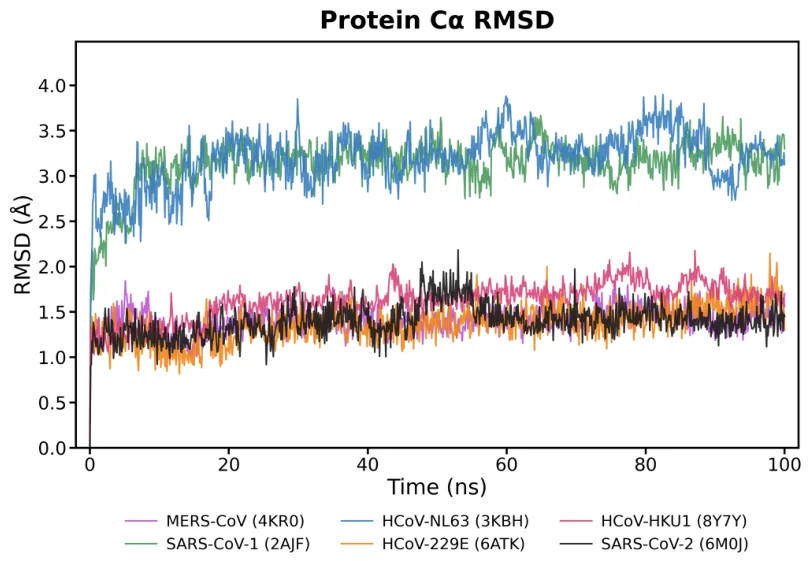
Protein backbone Cα root-mean-square deviation (RMSD) profiles for the six CKP-25 coronavirus spike complexes over 100 ns production simulations. All systems show stable convergence within the first 10–20 ns of trajectory. SARS-CoV-2 (6M0J, black) is shown as the experimentally validated reference system rather than as a sixth test target.

The structural stability of each protein–ligand complex was monitored via the Cα RMSD of the protein backbone throughout the 100 ns trajectory (Figure 3). All six systems followed a consistent pattern: an initial increase in Cα RMSD during approximately the first 10–20 ns, reflecting relaxation from the position-restrained equilibration state, followed by convergence to a stable plateau for the remainder of the simulation. Backbone plateaus were reproducible between replicates, with between-replicate standard deviations of 0.04–0.40 Å (Table 2).

**Figure 4:**
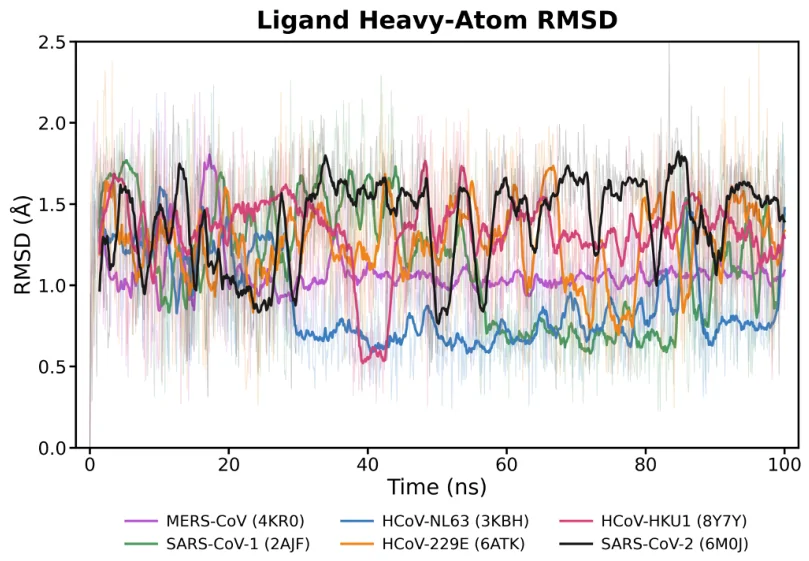
Ligand heavy-atom RMSD profiles of CKP-25 bound to the six coronavirus spike RBDs over 100 ns. Faint lines represent raw high-frequency data, while solid lines show the moving average (15-frame window), highlighting stable low-RMSD plateaus. SARS-CoV-2 (6M0J, black) is shown as the experimentally validated reference system rather than as a sixth test target.

**Figure 5:**
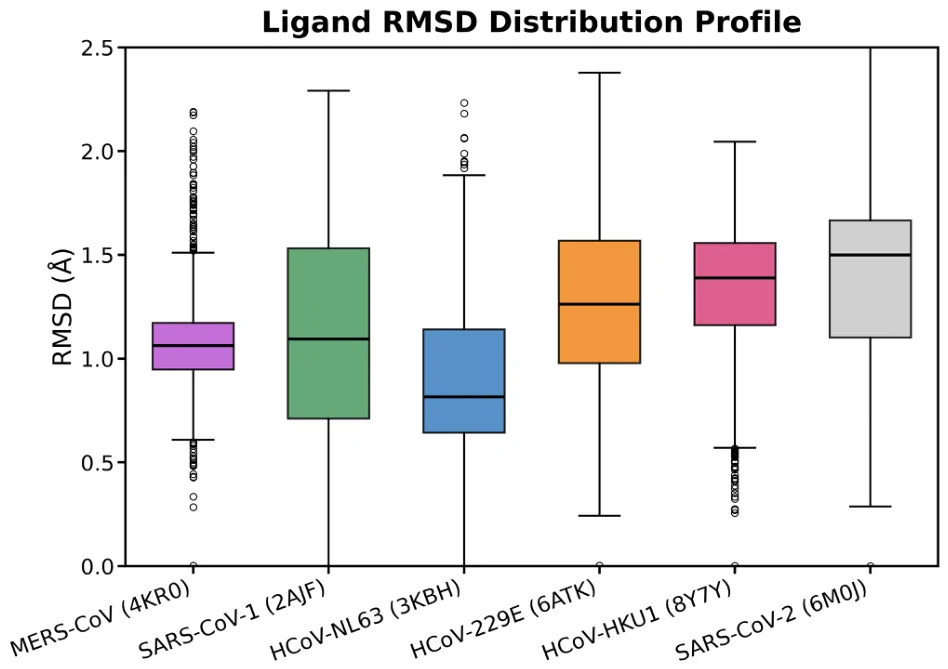
Distribution of ligand RMSD values across the six complexes over the production trajectories. Boxes show the interquartile range, center lines the median, and whiskers 1.5× the interquartile range; open circles denote frame-level outliers. Distributions are computed from the single representative replicate plotted in Figure 4, so their width reflects frame-to-frame fluctuation within one trajectory rather than reproducibility between replicates. SARS-CoV-2 (6M0J, grey) is included as the experimentally validated reference rather than as a sixth test target.

Ligand heavy-atom RMSD remained low in every system and in every replicate, with second-half plateaus between 1.00 and 2.06 Å (Figure 4 and Figure 5, summarized in Table 2). The most conformationally restrained environments were the HCoV-NL63 (1.00±0.32 Å) and SARS-CoV-1 (1.22±0.33 Å) complexes, and the least restrained was HCoV-HKU1 (2.06±0.77 Å), which also showed the largest variation between replicates.

### 2.4. Per-Residue Flexibility and Global Compactness

**Figure 6:**
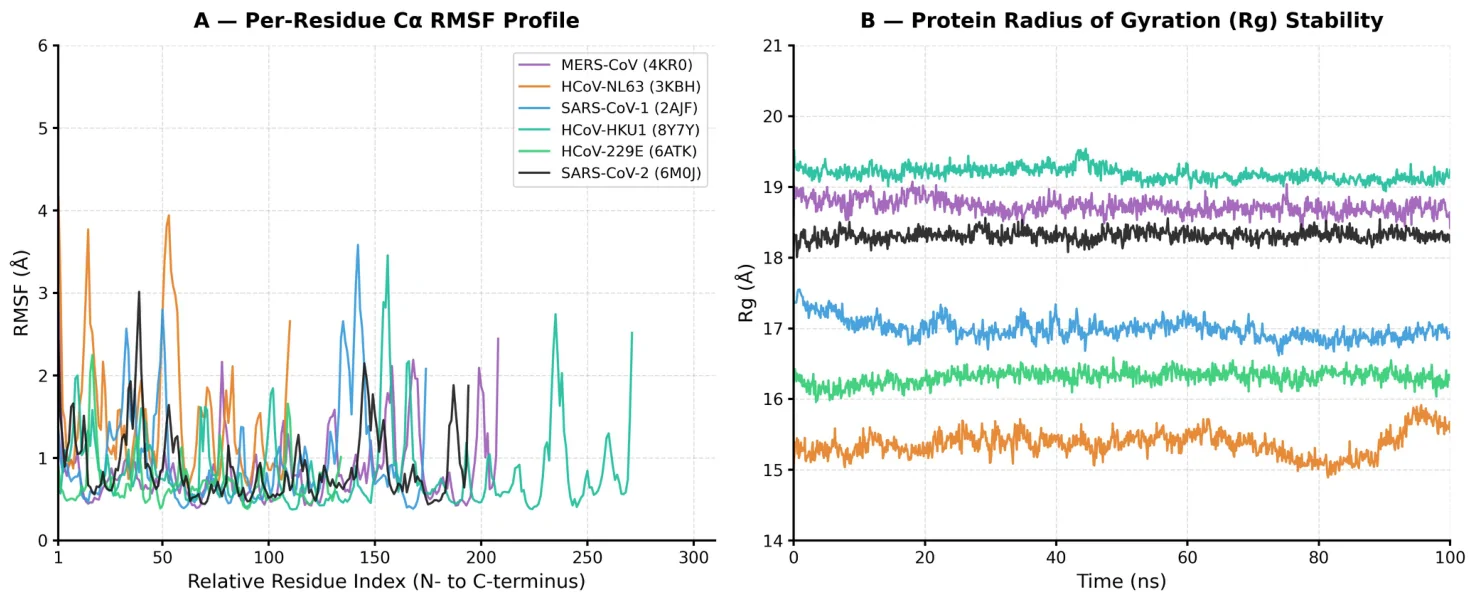
System dynamics and structural stability analysis for CKP-25 complexed with six human coronavirus spike RBDs. (**A**) Per-residue Cα root-mean-square fluctuation (RMSF) profiles aligned by relative residue index from N- to C-terminus, illustrating elevated flexibility in peripheral loop regions and comparatively lower flexibility around receptor-interface regions. (**B**) Time-series of the protein radius of gyration ($R_{g}$) over the 100 ns trajectories, showing stable global compactness across all simulated systems. SARS-CoV-2 (6M0J, black) is shown as the experimentally validated reference system rather than as a sixth test target.

Per-residue Cα RMSF profiles (Figure 6A) indicated that peripheral loop regions of the receptor-binding domains displayed elevated flexibility, whereas the receptor-interface regions occupied by CKP-25 generally exhibited comparatively lower fluctuations. This pattern is consistent with local stability of the ligand-bound interface during the production simulations.

Table 2 shows that the backbone Cα RMSD plateaus differ markedly between systems, with SARS-CoV-1 (3.03±0.28 Å) and HCoV-NL63 (3.45±0.33 Å) roughly twice those of the remaining four. Comparing the RMSF of the reproducible anchor residues with the domain-wide mean shows that this does not reflect destabilisation of the ligand-bound interface. For SARS-CoV-1, the anchor residues fluctuate by 0.70±0.00 Å against a domain mean of 1.04±0.05 Å, with the largest excursions confined to the peripheral Pro469–Leu472 loop (up to 3.6 Å). The elevated global RMSD is therefore driven by peripheral loop motion remote from the binding site. HCoV-NL63 requires a different explanation: it is by some margin the smallest construct in the panel (110 resolved residues, against 134–271 for the others), so a given physical displacement of its termini and loops contributes proportionally more to a domain-wide RMSD, and its domain-wide RMSF is correspondingly the highest of the six (1.35±0.15 Å). Its anchor residues remain comparatively restrained (1.12±0.10 Å).

Critically, this backbone flexibility does not propagate to the bound ligand. HCoV-NL63 gave the lowest ligand RMSD plateau of the entire panel (1.00±0.32 Å), the second-highest contact density (11.53±1.42 per frame), and retained all seven of its anchor residues in every replicate; SARS-CoV-1 likewise retained its four anchors throughout. The converse pattern is more informative: HCoV-HKU1 combines a modest backbone plateau (2.09±0.40 Å) with the only interface in the panel that fluctuates more than its own domain average (1.31±0.35 Å at the anchor residues against 0.93±0.09 Å domain-wide), and it is this system, rather than the two with elevated global RMSD, that shows the largest ligand RMSD (2.06±0.77 Å) and the widest spread in binding free energy.

The radius of gyration ($R_{g}$) was monitored to detect potential large-scale unfolding. The $R_{g}$ values remained stable with minimal fluctuation across all systems (Figure 6B), supporting the preservation of overall structural compactness throughout the simulations. The mean $R_{g}$ values over the production trajectories, averaged across the three replicates, were: MERS-CoV B-spike (18.76±0.05 Å), SARS-CoV-1 E-spike (17.13±0.13 Å), HCoV-229E E-spike (16.28±0.03 Å), HCoV-HKU1 A-spike (19.30±0.10 Å), HCoV-NL63 E-spike (15.42±0.12 Å), and the SARS-CoV-2 reference (18.39±0.08 Å). The between-replicate variation of at most 0.13 Å confirms that none of the domains, including the two with elevated backbone RMSD, underwent partial unfolding or loss of compactness.

### 2.5. Trajectory Contacts and Polar Interactions

**Figure 7:**
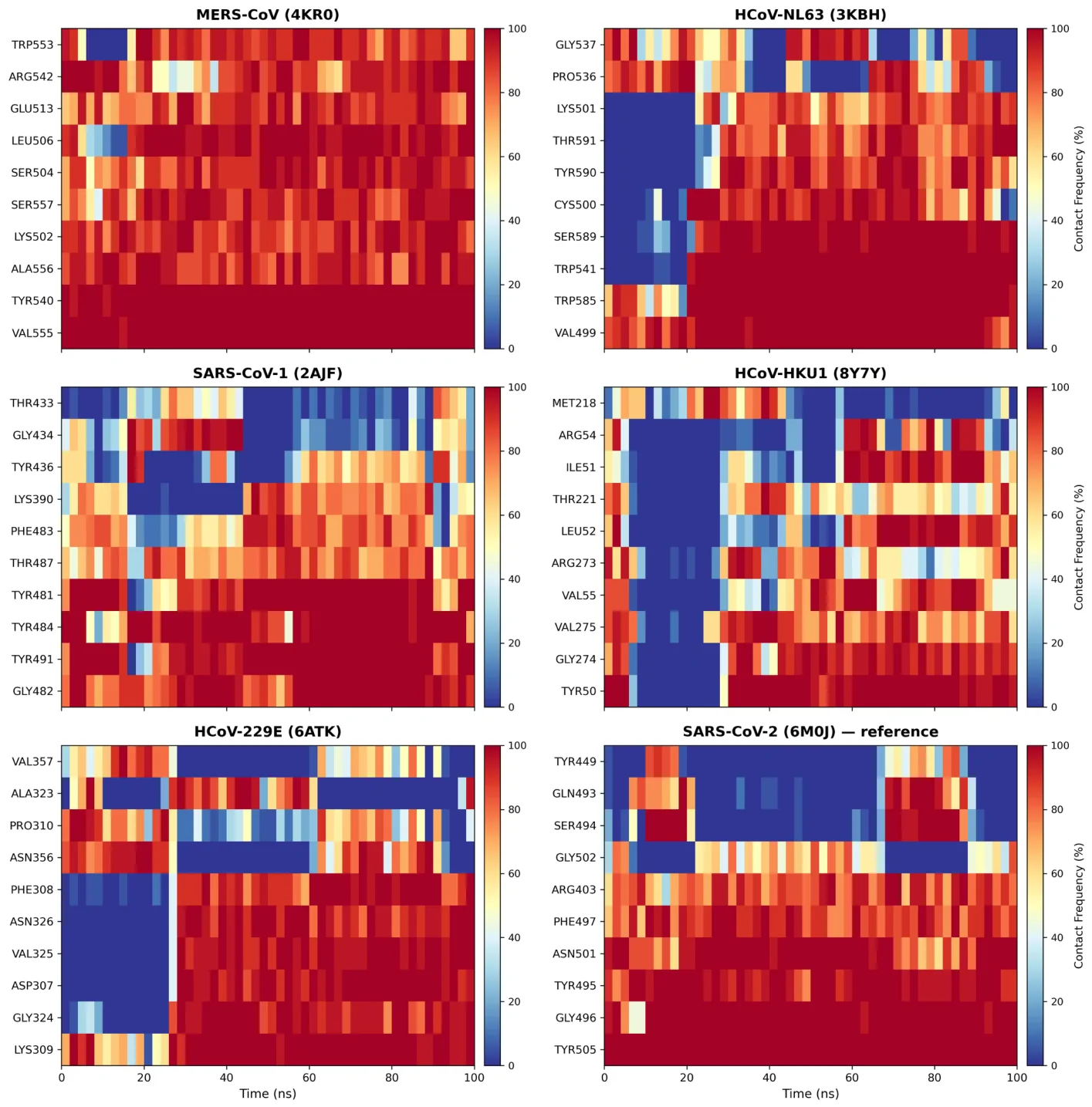
Residue-specific protein–ligand contact frequency heatmaps for CKP-25 complexed with the six coronavirus spike targets over the 100 ns MD trajectories. For each target, the top 10 interacting residues are displayed along the y-axis, with contact frequency calculated using a 4.0 Å distance cutoff and binned in 2 ns intervals. The color gradient from blue (low contact frequency) to red (high contact frequency) highlights persistent anchoring of CKP-25 against key aromatic, hydrophobic, and polar interface residues. Each panel shows a single representative replicate rather than an average over the three. This panel represents five of the six systems, in that its top-ranked residues recover every residue identified as a reproducible anchor in Section 2.5. HCoV-229E is the exception: the replicate shown is the one in which CKP-25 remained at the docked site, whereas it relocated in the other two, and none of the residues in its panel meets the reproducible-anchoring criterion. The apparent persistence of the HCoV-229E contacts is therefore specific to the replicate displayed and should not be read as stable occupancy of that interface. SARS-CoV-2 (6M0J) is shown as the experimentally validated reference system rather than as a sixth test target; its panel reproduces the previously reported π–π anchoring of CKP-25 on Tyr505.

**Figure 8:**
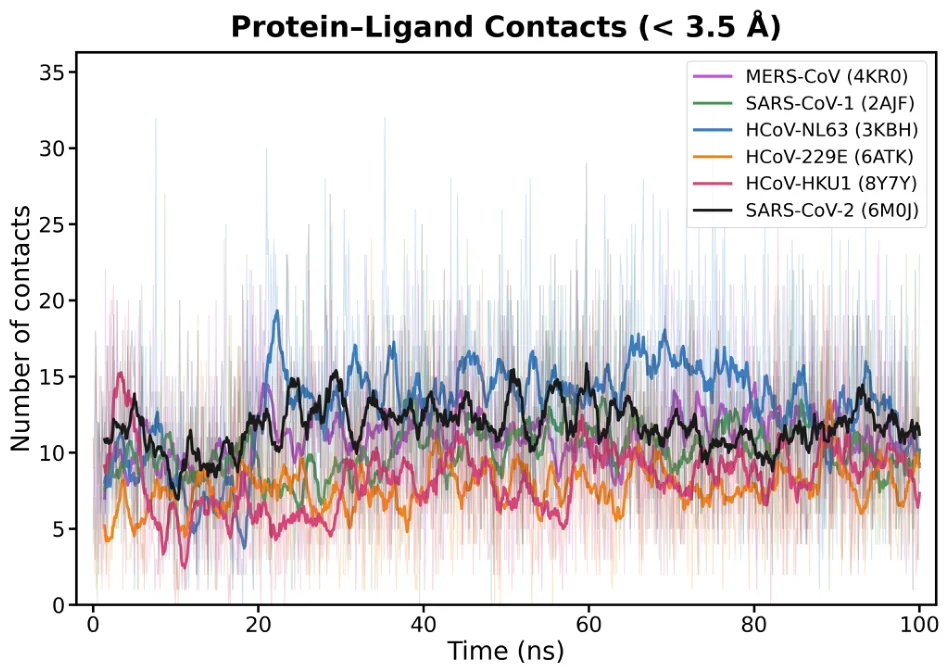
Protein–ligand heavy atom contact counts within 3.5 Å throughout the 100 ns MD trajectories. Faint lines indicate raw contact counts per frame, while solid lines represent the moving average using a 15-frame window, showing persistent close-contact interaction profiles across all six complexes. SARS-CoV-2 (6M0J, black) is shown as the experimentally validated reference system rather than as a sixth test target.

Total close protein–ligand contacts and polar contacts were analyzed separately to characterize the interaction profile of CKP-25 across the six coronavirus spike targets. residue-level contact frequencies were mapped using a 4.0 Å cutoff (Figure 7) while close heavy-atom contacts within 3.5 Å were tracked continuously throughout the trajectories (Figure 8). Averaged over three independent replicates, the SARS-CoV-2 reference complex maintained the highest and most reproducible interaction density (11.85±0.19 contacts per frame), followed by HCoV-NL63 (11.53±1.42) and MERS-CoV (10.39±0.61). SARS-CoV-1 (9.84±0.37), HCoV-229E (9.64±2.26), and HCoV-HKU1 (8.98±1.71) gave lower counts. The between-replicate spread is itself informative: MERS-CoV, SARS-CoV-1, and SARS-CoV-2 varied by fewer than 0.7 contacts per frame between runs, whereas HCoV-229E and HCoV-HKU1 varied by 1.7–2.3, indicating a less consistently defined interaction surface.

**Figure 9:**
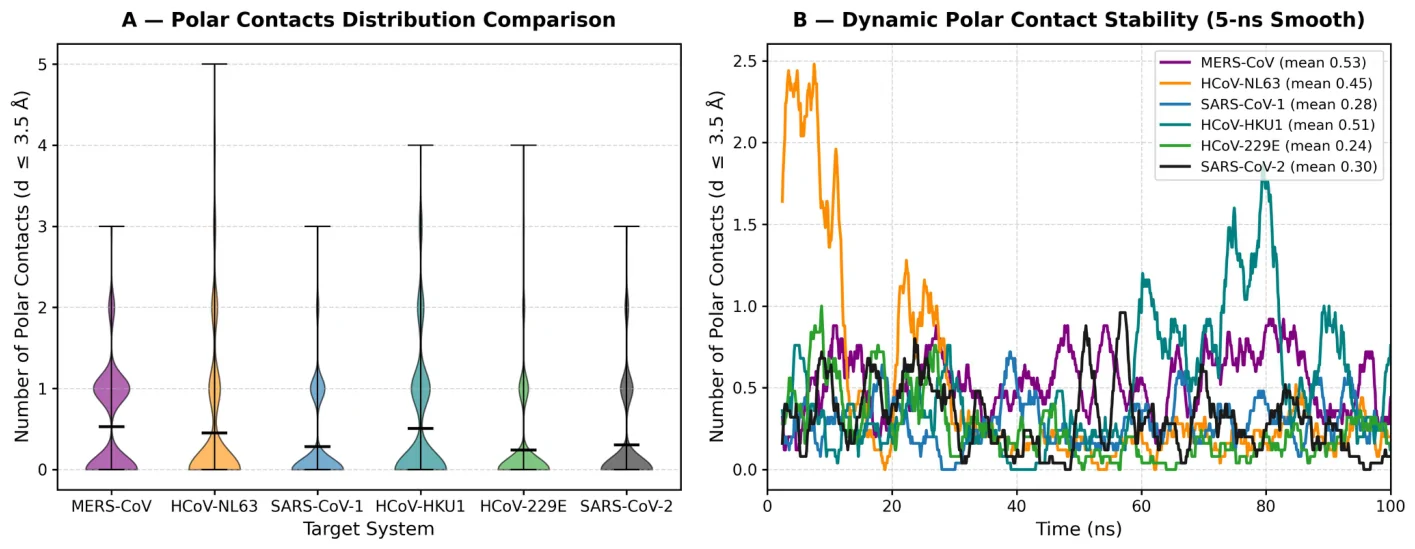
Analysis of protein–ligand polar contacts (d≤3.5 Å) for CKP-25 bound to six coronavirus spike receptor-binding domains. (**A**) Distribution profiles of polar contacts across the 100 ns trajectories represented as violin plots. The black horizontal bars denote the mean values, illustrating that the polar contacts are dynamic and fluctuate mainly between 0, 1, 2, and 3 contacts per frame because of the open-pocket nature of the binding sites. (**B**) Five-nanosecond moving-average time series showing that, although these polar interactions are transient, they are intermittently formed throughout the 100 ns simulation window. SARS-CoV-2 (6M0J, black) is shown as the experimentally validated reference system rather than as a sixth test target.

A detailed analysis of polar contacts (d≤3.5 Å) between the polar atoms of CKP-25 and the target spike proteins revealed a highly dynamic interaction network (Figure 9). Because the host-receptor interface on the spike protein represents a solvent-exposed, open binding pocket, the ligand’s polar groups undergo continuous micro-conformational rotations. Consequently, strict geometric hydrogen bonds (requiring strict donor–acceptor angles) are rare, while broader polar contacts fluctuate rapidly. This dynamic behavior is reflected in the low but persistent average polar-contact counts, ranging from 0.23±0.05 contacts per frame for SARS-CoV-1 to 0.49±0.09 for HCoV-HKU1, with the SARS-CoV-2 reference at 0.42±0.14 (Figure 9A). The moving averages (Figure 9B) indicate that these polar interactions are intermittently formed throughout the 100 ns simulations. Rather than relying on a rigid, static hydrogen-bond network, CKP-25 appears to maintain stability primarily through shape-complementary packing, with transient polar contacts acting as secondary stabilizing interactions.

Residues maintaining contact (heavy atoms <4.0 Å) for >50% of the production window in each of the three independent replicates represent reproducible structural anchors. Percentages are the mean across replicates:

- **MERS-CoV (4KR0)**: VAL555 (100%), TYR540 (99%), ALA556 (94%), LYS502 (90%), SER504 (86%), SER557 (85%), GLU513 (84%), ARG542 (78%).
- **SARS-CoV-1 (2AJF)**: TYR491 (81%), GLY482 (77%), TYR481 (76%), TYR484 (74%).
- **HCoV-NL63 (3KBH)**: TRP585 (92%), TRP541 (83%), CYS500 (83%), SER589 (80%), TYR590 (65%), THR591 (63%), LYS501 (55%).
- **HCoV-HKU1 (8Y7Y)**: TYR50 (86%), GLY274 (83%), VAL55 (76%), VAL275 (64%).
- **SARS-CoV-2 (6M0J, reference)**: TYR505 (92%), GLY496 (91%), TYR495 (89%), ASN501 (80%), ARG403 (79%), PHE497 (77%).
- **HCoV-229E (6ATK)**: no residue satisfied this criterion (see below).

HCoV-229E was the single exception in the panel. In the first replicate, CKP-25 remained at the docked receptor-recognition site (LYS309, GLY324, ASP307, VAL325, ASN326, PHE308), but in the two remaining replicates, it relocated to distinct alternative surface sites (TYR350, SER363, ILE364 in one; ARG359, GLY358, ASN356 in the other), sharing no residue with the initial site or with each other. The compound therefore remained associated with the HCoV-229E spike but did not stably occupy its receptor-recognition interface. This behavior is consistent with the other measures obtained for this system, which gave the lowest CNN pose score of the panel (0.692), the lowest fraction of buried ligand surface, and the largest replicate-to-replicate variation in contact count (±2.26 per frame). Because the three replicates do not sample a common binding mode, the MM-GBSA value reported for HCoV-229E in Section 2.6 should be interpreted as an average over distinct surface interactions rather than as an estimate for a single defined site. This persistent engagement supports a binding mode dominated by shape-complementary van der Waals interactions against hydrophobic/aromatic interface residues. However, because contact frequency measures only structural proximity rather than interaction quality, a high contact count does not always translate directly to stronger thermodynamic stability. To bridge this structural analysis with thermodynamic binding energetics, we carried out MM-GBSA free energy calculations (Section 2.6).

### 2.6. Thermodynamic Binding Free Energy (MM-GBSA)

To investigate the thermodynamic driving forces behind CKP-25’s binding across the six coronavirus spike targets, we performed Molecular Mechanics–Generalized Born Surface Area (MM-GBSA) calculations. The total binding free energy ($\Delta G_{\mathrm{bind}}$) was decomposed into individual physical energy terms according to Equation  (1):

(1) $$\Delta G_{\mathrm{bind}}=\Delta E_{\mathrm{vdw}}+\Delta E_{\mathrm{el}}+\Delta G_{\mathrm{GB}}+\Delta G_{\mathrm{surf}}$$

where $\Delta E_{\mathrm{vdw}}$ is the van der Waals interaction energy, $\Delta E_{\mathrm{el}}$ is the electrostatic interaction energy, $\Delta G_{\mathrm{GB}}$ is the polar solvation free energy (modeled using the Generalized Born implicit-solvent model with a salt concentration of 0.150 M; the setting igb=2 selects the modified Onufriev–Bashford–Case pairwise GB variant [16], used here with mbondi2 intrinsic Born radii, which is the combination recommended for protein–ligand systems in AmberTools), and $\Delta G_{\mathrm{surf}}$ is the non-polar solvation cavity term calculated from the solvent-accessible surface area (SASA) using the LCPO algorithm. The calculations were averaged over 100 frames extracted at 500 ps intervals from the stable 50–100 ns production window of the MD trajectories. The resulting energy components and total binding free energies are summarized in Table 3.

The thermodynamic decomposition reveals that the binding of CKP-25 to all six target interfaces is dominated by non-polar interactions. The sum of the non-polar terms ($\Delta E_{\mathrm{vdw}}+\Delta G_{\mathrm{surf}}$) ranges from −31.44 kcal/mol for SARS-CoV-1 to −43.07 kcal/mol for MERS-CoV, representing close shape-complementary packing and hydrophobic surface burial at the interface. This component analysis also explains why contact count and binding free energy do not track one another: HCoV-NL63 maintains more contacts per frame than MERS-CoV (11.53±1.42 vs. 10.39±0.61), yet MERS-CoV gives the more favorable mean binding free energy. MERS-CoV achieves both better van der Waals packing ($\Delta E_{\mathrm{vdw}}=-38.89$ vs. −35.16 kcal/mol) and greater hydrophobic burial ($\Delta G_{\mathrm{surf}}=-4.18$ vs. −3.50 kcal/mol) at a comparable desolvation cost ($\Delta G_{\mathrm{GB}}=11.50$ vs. 11.25 kcal/mol) and therefore forms fewer but energetically higher-quality contacts. We note, however, that the difference between these two systems (2.91 kcal/mol) is smaller than the replicate-to-replicate spread of either and should not be over-interpreted.

Conversely, the net polar contribution ($\Delta E_{\mathrm{el}}+\Delta G_{\mathrm{GB}}$) is unfavorable across all targets, contributing positive free energies between +6.71 kcal/mol (HCoV-229E) and +8.49 kcal/mol (MERS-CoV). This discouraging polar contribution arises because the energetic cost of stripping water molecules from the ligand and pocket polar groups (polar desolvation, $\Delta G_{\mathrm{GB}}$) outweighs the direct electrostatic and hydrogen-bonding interactions formed ($\Delta E_{\mathrm{el}}$) at these solvent-exposed interfaces. These findings provide quantitative, physical evidence that CKP-25 is held at these interfaces primarily through shape-complementary hydrophobic/aromatic packing, while polar contacts serve as secondary, fluctuating stabilizers that must overcome substantial desolvation penalties.

Inclusion of the SARS-CoV-2 reference system allows these values to be placed on an interpretable scale. CKP-25 gave −29.15±3.46 kcal/mol at the interface where it has demonstrated antiviral activity, and none of the five test targets differed from this value by more than the combined replicate scatter. MERS-CoV and HCoV-NL63 returned more favorable means (−34.57 and −31.66 kcal/mol), and SARS-CoV-1 a less favorable one (−24.28 kcal/mol), but in every case, the separation from the reference falls within the spread of the individual replicates. The defensible conclusion is therefore not a ranking among the targets but that CKP-25 engages all six receptor-recognition interfaces with binding free energies indistinguishable from that obtained for the one system in which it is experimentally active.

The between-replicate standard deviations are themselves informative. MERS-CoV (±3.41), HCoV-NL63 (±3.54), SARS-CoV-1 (±1.46), and the SARS-CoV-2 reference (±3.46) reproduced to within a few kcal/mol across independent trajectories, confirming that the protocol yields consistent estimates when the ligand occupies a single, well-defined site. HCoV-HKU1 (±10.61) and HCoV-229E (±7.18) did not. For HCoV-229E, this is explained by relocation of the ligand between replicates (Section 2.5); for HCoV-HKU1, the ligand retained its anchor residues in all three runs, so the variation instead reflects differing depths of burial within the same shallow pocket, consistent with the large replicate spread in its van der Waals term ($\Delta E_{\mathrm{vdw}}=-31.74\pm 10.92$ kcal/mol). Free-energy estimates for these two systems should therefore be treated as less firmly determined than those for the remaining four.

## 3. Discussion

The findings of this study highlight the value of combining molecular docking with explicit-solvent molecular dynamics (MD) simulations to computationally evaluate the binding stability of small molecules at coronavirus spike receptor-recognition interfaces. While static docking frameworks such as GNINA are useful for rapid pose generation and virtual screening, they cannot fully capture the structural plasticity and relaxation of the target binding interface. This limitation is illustrated by the discrepancy between the initial GNINA-reported docking affinities and the final MD/MM-GBSA binding-energy estimates. GNINA predicted HCoV-HKU1 to have the best docking affinity (−6.76 kcal/mol), followed by MERS-CoV (−6.61 kcal/mol). After 100 ns MD and MM-GBSA profiling, however, HCoV-HKU1 gave one of the least favorable and by far the least reproducible binding free energies of the panel (−28.12±10.61 kcal/mol), whereas MERS-CoV returned the most favorable mean (−34.57±3.41 kcal/mol). The clearest illustration is provided by the reference system itself: SARS-CoV-2, the only interface at which CKP-25 is experimentally active, ranked fifth of six by docking affinity (−6.03 kcal/mol) yet returned a mid-range binding free energy that none of the five test targets differed from beyond replicate scatter. A static docking score alone would therefore have mis-ordered the panel with respect to the one target whose behavior is independently known, which underlines the value of dynamic simulation as a complementary step after molecular docking.

A key structural finding is that CKP-25 achieves stable binding by anchoring within hydrophobic and aromatic receptor-interface environments across diverse and pre-exposed coronavirus spike proteins. Contact-frequency profiling identified a persistent reliance on shape-complementary van der Waals packing against aromatic and hydrophobic interface residues, including TYR540 in MERS-CoV, TRP541 and TRP585 in HCoV-NL63, TYR481, TYR484 and TYR491 in SARS-CoV-1, TYR50 in HCoV-HKU1, and TYR505, TYR495 and PHE497 in the SARS-CoV-2 reference. Each of these residues met the anchoring criterion independently in all three replicates, so the motif is reproducible rather than an artefact of a single trajectory. Its recovery in the reference system is particularly informative: Tyr505 was previously identified as the residue maintaining CKP-25 at the SARS-CoV-2 interface using a different force field [11], and it emerged here as the highest-frequency anchor (92% across replicates) without being targeted. Although the exact residues differ among viruses, these interaction patterns suggest that CKP-25 can exploit functionally analogous receptor-interface features across divergent human coronaviruses. Because receptor-recognition interfaces are directly involved in host-cell attachment, small molecules that occupy these regions may provide a strategy for interfering with viral entry. Nevertheless, the extent to which these computationally identified interface anchors are mutationally constrained requires further experimental and evolutionary validation.

This structural anchoring is further characterized by the dynamic behavior of polar contacts. While some small-molecule inhibitors rely on rigid, high-occupancy hydrogen bonds, CKP-25 appears to maintain stable binding primarily through shape-complementary packing, supported by transient polar contacts in solvent-exposed, open interface regions. In these regions, polar groups are subject to solvent competition from surrounding water molecules, which can continually form and break hydrogen bonds with both the ligand and the receptor [17,18]. Since CKP-25 is predominantly hydrophobic and contains only a small number of polar atoms, the primary driver of its interaction profile across these interfaces is likely driven by hydrophobic/aromatic packing. The auxiliary polar contacts fluctuate between 0 and 3 contacts per frame as the ligand adapts to the open interface, meaning they are transient rather than static. Their intermittent re-formation throughout the 100 ns trajectories suggests that they provide secondary electrostatic stabilization without requiring a rigid hydrogen-bond network. This dynamic polar engagement, combined with persistent hydrophobic and aromatic contacts, supports CKP-25’s stable interaction profile across the coronavirus spike targets at which it remained bound.

This decomposition carries a practical implication for optimizing the scaffold. The net polar contribution is unfavorable at every interface examined (+6.71 to +8.49 kcal/mol), yet CKP-25 presents no hydrogen-bond donors and a small polar surface (TPSA 27.1 ${Å}^{2}$; calculated logP 4.25). The penalty therefore arises mainly from desolvating the pocket rather than the ligand, so further polar substituents would be expected to increase it unless the resulting hydrogen bonds were geometrically well optimized—difficult at interfaces this open and solvent-exposed, as the low and transient polar-contact counts indicate. Enlarging the buried non-polar surface is the more promising route, though this is bounded by an already high logP. Substitutions that extend aromatic contact without adding appreciable lipophilicity, exploiting the conserved tyrosine- and tryptophan-rich environments identified here, are the rational starting point for a medicinal-chemistry campaign.

Replication changed how the thermodynamic data can be read. Averaged over three trajectories, the six systems span −24.28 to −34.57 kcal/mol, but between-replicate standard deviations range from ±1.46 to ±10.61 kcal/mol, and no target separates from the SARS-CoV-2 reference by more than the combined scatter. The stratification into distinct binding-energy tiers that single trajectories had suggested is therefore not supported. At the four test interfaces where CKP-25 remained at the receptor-recognition site, it binds with free energies indistinguishable from that obtained where the compound is experimentally active, MERS-CoV and HCoV-NL63 giving the most favorable means. Reproducibility is the more discriminating observation: MERS-CoV, HCoV-NL63, SARS-CoV-1 and the reference each reproduced to within a few kcal/mol, whereas HCoV-HKU1 and HCoV-229E did not, for the distinct reasons set out in Section 2.5 and Section 2.6. Furthermore, these free-energy values should be interpreted strictly as relative computational estimates rather than absolute experimental binding free energies. Because MM-GBSA does not include rigorous conformational entropic penalties (−TΔS), the calculated absolute free energies may be over-encouraging; therefore, direct conversion to experimental dissociation constants (Kd) would be inappropriate.

The behavior of HCoV-229E warrants separate comment. CKP-25 occupied its docked receptor-recognition site in only one of three replicates, relocating in the other two to alternative surface sites that shared no residues with the initial site or with each other. Rather than treating this as a failed simulation, we interpret it as a property of the interface: HCoV-229E gave the lowest CNN pose score of the panel, the smallest fraction of buried ligand surface, and the largest replicate-to-replicate variation in contact count. Taken together, these indicate a shallow and poorly enclosed pocket that offers a low barrier to relocation, and they argue against including HCoV-229E among the interfaces that CKP-25 can be expected to occupy stably. A negative result of this kind is more useful than an indiscriminate positive one, since a compound that appeared to bind every interface tested would be difficult to distinguish from one binding none of them specifically.

Taken together, the present results establish computationally that CKP-25 occupies the receptor-recognition interface of four of the five human coronaviruses examined—MERS-CoV, HCoV-NL63, SARS-CoV-1 and HCoV-HKU1—reproducibly across independent replicates, through a conserved aromatic and hydrophobic anchoring motif, and with predicted binding free energies that do not differ from those obtained at the SARS-CoV-2 interface at which the compound is experimentally active. Three separate lines of evidence converge on this conclusion: retention of the ligand at the intended site in every replicate of those four systems, recovery of the same anchor residues in each independent run, and energetic equivalence to a validated reference carried through the identical protocol. We emphasize that this is a computational prediction rather than a demonstration of antiviral activity. The simulations establish that CKP-25 can occupy these interfaces stably and with comparable energetics; they do not establish that such occupancy is sufficient to block receptor engagement or viral entry in cells, which remains to be tested experimentally.

While this study provides computational evidence that CKP-25 can occupy these interfaces, several limitations must be acknowledged. First, the 100 ns simulation window supports local pocket stability but cannot fully capture larger-scale conformational changes associated with spike protein activation or viral membrane fusion. The relocation events observed for HCoV-229E, and in one replicate of the SARS-CoV-2 reference, indicate that this window is nonetheless long enough for a small, lipophilic ligand to sample alternative surface sites on these domains. Second, the simulations were performed on isolated spike receptor-binding regions in explicit water, omitting the lipid envelope, full spike-trimer context, and the extensive glycan shield that covers the spike protein in vivo. Although receptor-binding interfaces must remain at least partially accessible to allow host interaction, surrounding glycans and higher-order spike architecture could influence ligand access and binding stability. Consequently, future work should evaluate CKP-25 in the context of fully glycosylated spike trimers and validate these computational predictions through in vitro assays, such as surface plasmon resonance (SPR) or biolayer interferometry binding studies, as well as pseudotyped-virus entry or neutralization assays.

## 4. Materials and Methods

### 4.1. Target Selection and Receptor-Binding Hotspot Identification

Experimentally determined structures of human coronavirus spike receptor-binding regions in complex with their respective host receptors or entry mediators were retrieved from the Protein Data Bank (PDB). Targets were selected to represent phylogenetically diverse alpha- and betacoronaviruses, distinct host-receptor usage, and structurally resolved receptor-recognition interfaces suitable for hotspot mapping. The selected systems were:

- **MERS-CoV B-spike**: receptor-binding domain complexed with human dipeptidyl peptidase 4 (DPP4) at 2.70 Å resolution (PDB ID: 4KR0) [19].
- **SARS-CoV-1 E-spike**: receptor-binding domain complexed with human angiotensin-converting enzyme 2 (ACE2) at 2.90 Å resolution (PDB ID: 2AJF) [20].
- **HCoV-NL63 E-spike**: receptor-binding domain complexed with human ACE2 at 3.31 Å resolution (PDB ID: 3KBH) [21].
- **HCoV-229E E-spike**: receptor-binding domain complexed with human aminopeptidase N (APN) at 3.50 Å resolution (PDB ID: 6ATK) [22].
- **HCoV-HKU1 A-spike**: cryo-electron microscopy structure of the spike receptor-binding region complexed with human transmembrane serine protease 2 (TMPRSS2) at 3.24 Å resolution (PDB ID: 8Y7Y) [15].
- **SARS-CoV-2 E-spike (reference system)**: receptor-binding domain complexed with human ACE2 at 2.45 Å resolution (PDB ID: 6M0J) [23].

SARS-CoV-2 was included as an experimentally validated reference rather than as an additional test target. CKP-25 was originally identified against this interface and reduces SARS-CoV-2 viral titres in cell culture [11], so it provides the only system in the panel for which the compound has demonstrated antiviral activity. It was prepared, docked, simulated, and analyzed with exactly the same protocol as the five test targets and therefore calibrates the values obtained for them.

For each complex, the selected viral spike chain was extracted for subsequent docking and molecular dynamics simulations, while the coordinates of the human host partner were removed. Before removal, the host-partner coordinates were used to define the native receptor-recognition hotspot on the viral spike. The hotspot was defined as the set of spike residues containing at least one heavy atom within 4.0 Å of a host-receptor heavy atom, consistent with a commonly used structural cutoff for identifying non-covalent contacts at protein–protein interfaces [24]. The resulting target-specific hotspot residues were used to determine the center and dimensions of the focused docking grid for CKP-25.

### 4.2. Molecular Docking Protocol

Initial binding poses of CKP-25 against the six target spike RBDs were generated using GNINA 1.3, a state-of-the-art molecular docking framework that integrates conventional empirical scoring functions with a deep learning-based 3D convolutional neural network (CNN) for pose scoring and affinity prediction [25]. GNINA 1.3 was selected because its learned CNN scoring functions are designed to capture shape and chemical complementarity in protein–ligand complexes, supporting its use for evaluating CKP-25 binding at solvent-exposed receptor-recognition interfaces.

To perform targeted docking, a focused 3D bounding box (exhaustiveness = 8) was defined separately for each of the six viral spike targets. The box was centered precisely over the identified host-interaction hotspot residues of the isolated spike RBD rather than using a single uniform box or a global blind search across the entire glycoprotein. Because the six spike RBDs exhibit highly divergent sequence alignments and interface topologies, the box dimensions were custom-tailored for each target to encompass the coordinates of their respective hotspot residues plus a default buffer of 4.0 Å on all six sides. This produced target-specific focused docking boxes centered on the native receptor-recognition region, allowing CKP-25 to explore conformations capable of occupying the host-interaction interface without imposing a single uniform box size across structurally distinct spike targets. The resulting box centers and dimensions are reported in full in Table 4 in the coordinate frame of the corresponding PDB entry. Additional docking-box coordinate details are provided in Supplementary Table S1 and its accompanying CSV file.

Importantly, this localized docking approach does not bias the final results: the grid box is used solely to generate the initial starting coordinates for the docking pose. The subsequent 100 ns explicit-solvent MD simulations are completely unrestrained, allowing the ligand and spike protein to undergo conformational changes, relax, or dissociate. Thus, the thermodynamic stability and contact frequencies reported in this study reflect the unrestrained physical behavior of the complexes, independent of the initial docking box boundaries. Poses for each target were ranked primarily by their CNN pose score (which serves as the primary metric in GNINA for structural correctness) while applying a Vina-predicted binding affinity of <−5.0 kcal/mol as a secondary thermodynamic filter. The highest-ranked pose that satisfied this Vina filter was selected and extracted as the starting structure for the subsequent MD simulations.

### 4.3. Molecular Dynamics Simulations

All-atom MD simulations were performed using GROMACS 2025.4 with GPU acceleration [26]. All simulations were performed on an NVIDIA RTX 3090 GPU with 24 GB of memory.

System preparation. The protein topology for each spike domain was generated with gmx pdb2gmx using the AMBER99SB-ILDN force field [27] and the TIP3P explicit water model [28]. AMBER99SB-ILDN was selected because of its improved side-chain torsion parameters, which enhance accuracy for side-chain conformation and packing at protein–protein interfaces. This choice was additionally dictated by internal parameter consistency across the whole pipeline: the ligand was described with GAFF2 and AM1-BCC charges, which were developed and validated for use with AMBER-family protein force fields, and the end-state free-energy step relies on the AMBER-native Generalized Born model (igb=2, mbondi2 radii) implemented in AmberTools MMPBSA.py. Adopting a CHARMM-family protein force field would therefore have required the simultaneous re-parametrization of the ligand with CGenFF and reconstruction of the MM-GBSA stack, altering two independent components of the protocol rather than one. Ligand topology and partial charges were generated with ACPYPE [29] (interfacing with AmberTools [30] antechamber; GAFF2 parameters; gas-phase AM1-BCC charges; net charge = 0). Although AM1-BCC does not account for explicit polarization effects in highly aromatic systems, it is heavily validated to reproduce HF/6-31G* electrostatic potentials, providing a highly reliable and computationally efficient charge distribution for GAFF-family small-molecule parametrization. The protein–ligand complex was assembled and placed in a dodecahedral periodic box with a minimum 1.2 nm solute-to-wall distance (gmx editconf), solvated with TIP3P water (gmx solvate) and neutralized by the addition of ${\mathrm{Na}}^{+}$ and ${\mathrm{Cl}}^{-}$ counterions (gmx genion).

Energy minimization; Each solvated, charge-neutralized system was subjected to steepest-descent energy minimization (up to 50,000 steps; convergence criterion: $F_{\max}<1000$ kJ ${\mathrm{mol}}^{-1}$ ${\mathrm{nm}}^{-1}$) using Particle Mesh Ewald (PME) electrostatics and a Verlet pair-list cutoff scheme ($r_{\mathrm{coulomb}}=r_{\mathrm{vdw}}=1.0$ nm).

Equilibration. Two sequential equilibration phases were performed with harmonic position restraints on all protein and ligand heavy atoms:

1. NVT (100 ps; 50,000 steps × 2 fs): temperature maintained at 300 K using the V-rescale thermostat ($\tau_{T}=0.1$ ps) applied separately to the protein–ligand complex and solvent/ion groups.
2. NPT (100 ps; 50,000 steps × 2 fs): pressure maintained at 1.0 bar using the Parrinello–Rahman barostat ($\tau_{P}=2.0$ ps; compressibility = $4.5\times 10^{-5}$ ${\mathrm{bar}}^{-1}$; isotropic coupling); V-rescale thermostat retained.

Production MD; Unrestrained production simulations were run for 100 ns, corresponding to 50,000,000 steps with a 2 fs timestep, for each of the six protein–ligand complexes under the NPT ensemble. Temperature control was maintained with the V-rescale thermostat at 300 K ($\tau_{T}=0.1$ ps). Pressure was controlled at 1.0 bar with the Parrinello–Rahman barostat ($\tau_{P}=2.0$ ps; compressibility = $4.5\times 10^{-5}$ ${\mathrm{bar}}^{-1}$). Long-range electrostatics were treated by PME (Fourier grid spacing: 0.12 nm). Van der Waals interactions were truncated at 1.0 nm with analytic long-range dispersion corrections applied to both energy and pressure. All bonds involving hydrogen atoms were constrained using the LINCS algorithm. Coordinates were written every 10 ps to compressed XTC trajectory files. The 100 ns simulation length is consistent with established literature for evaluating local pocket occupancy and ligand stability at spike receptor-binding interfaces [11]. The initial 200 ps restrained equilibration, consisting of 100 ps under the NVT ensemble followed by 100 ps under the NPT ensemble, was used to relax the solvent and establish the target temperature and density. The complete 100 ns production trajectories were examined for structural stability, ligand retention, and protein–ligand contact persistence. For the end-state MM-GBSA calculations, the first 50 ns of each production trajectory were treated as a relaxation period, and 100 frames were extracted at 500 ps intervals from the equilibrated 50–100 ns window.

Replicate simulations; To assess the reproducibility of the structural and thermodynamic results, each of the six protein–ligand complexes was simulated in three fully independent 100 ns replicates, giving eighteen production trajectories in total. Replicates were generated by repeating the complete preparation and simulation protocol from the same docked starting pose, so that the initial coordinates, topology, force field, box geometry, and salt concentration were identical in every case. The replicates differ in the randomly generated initial atomic velocities (gen-vel = yes, gen-seed = −1, drawn independently at each NVT grompp step), in the stochastic seed of the V-rescale thermostat (ld-seed = −1), and in the placement of the neutralizing counterions by gmx genion. The values quoted in the Results are the mean over the three replicates, and the associated uncertainties are the between-replicate standard deviations rather than the frame-to-frame fluctuations within a single trajectory. Expanded MD statistics, complete simulation parameters, and replicate-specific random seeds are provided in Supplementary Tables S2, S3, and S7, respectively.

### 4.4. Trajectory Analysis and Free Energy Calculations

Structural analyses were performed on the 100 ns production trajectories. Prior to analysis, each trajectory was re-imaged to remove periodic-boundary artifacts and centered on the protein–ligand solute using gmx trjconv. Protein C_α_ root-mean-square deviation (RMSD), C_α_ root-mean-square fluctuation (RMSF), radius of gyration ($R_{g}$), ligand heavy-atom RMSD, and protein–ligand contacts were computed using MDTraj [31]. Ligand heavy-atom RMSD was computed after optimal superposition of the ligand heavy atoms onto their conformation in the reference frame. Close protein–ligand contacts were defined as pairs of heavy atoms within 3.5 Å. Polar contacts were defined as contacts between ligand and protein polar heavy atoms within 3.5 Å, whereas residue-specific contact frequencies were calculated using a 4.0 Å heavy-atom cutoff [24]. Additional residue-contact rankings, per-frame MM-GBSA energies, and per-replicate energy components are provided in Supplementary Tables S4–S6.

## 5. Conclusions and Limitations

By combining targeted deep learning-guided molecular docking with triplicate all-atom explicit-solvent molecular dynamics simulations, we computationally show that CKP-25 occupies the receptor-recognition interfaces of MERS-CoV, HCoV-NL63, SARS-CoV-1 and HCoV-HKU1 reproducibly, engaging the same aromatic and hydrophobic anchor residues in every independent replicate. Including SARS-CoV-2 as an experimentally validated reference places these results on an interpretable scale: the pipeline recovered the previously reported Tyr505 anchoring of CKP-25 at that interface using an independent force field, and none of the test targets differed from the reference in predicted binding free energy by more than the replicate-to-replicate scatter. HCoV-229E was the exception, with the ligand relocating from its docked site in two of three replicates, consistent with the shallowest and least enclosed pocket in the panel. These findings highlight the importance of replicate dynamic simulations as a validation step after static docking and indicate CKP-25 as a scaffold that potentially binds the receptor-recognition interface of diverse human coronaviruses. Experimental studies are required to validate the predicted binding behavior and to test whether occupancy of this pre-exposed receptor/entry-mediator site interferes with receptor engagement. As future work, we aim to measure CKP-25 binding to the four predicted targets using in vitro assays and to assess its antiviral activity against the corresponding viruses in cell culture.

## Figures and Tables

**Table 1:** GNINA docking results for CKP-25. Both the Vina-predicted affinity and the CNN pose score of the selected starting pose are reported; poses were ranked primarily by CNN pose score, which estimates structural correctness rather than affinity. SARS-CoV-2 (6M0J) is the experimentally validated reference system.

System (PDB)	Vina Affinity (kcal/mol)	CNN Pose Score
HCoV-HKU1 A-spike (8Y7Y)	−6.76	0.787
MERS-CoV B-spike (4KR0)	−6.61	0.763
HCoV-NL63 E-spike (3KBH)	−6.32	0.759
SARS-CoV-1 E-spike (2AJF)	−6.18	0.724
SARS-CoV-2 E-spike (6M0J)	−6.03	0.739
HCoV-229E E-spike (6ATK)	−5.93	0.692

**Table 2:** MD stability summary for all six CKP-25 coronavirus spike complexes. Values are the mean ± standard deviation of the 50–100 ns plateau across three independent replicates. SARS-CoV-2 (6M0J) is the experimentally validated reference system.

System (PDB)	Protein Cα RMSD	Ligand RMSD
	Plateau (Å)	Plateau (Å)
MERS-CoV B-spike (4KR0)	1.39±0.04	1.18±0.17
SARS-CoV-1 E-spike (2AJF)	3.03±0.28	1.22±0.33
HCoV-229E E-spike (6ATK)	1.29±0.18	1.36±0.11
HCoV-HKU1 A-spike (8Y7Y)	2.09±0.40	2.06±0.77
HCoV-NL63 E-spike (3KBH)	3.45±0.33	1.00±0.32
SARS-CoV-2 E-spike (6M0J)	1.63±0.17	1.48±0.17

**Table 3:** MM-GBSA thermodynamic binding free energy components (kcal/mol) for CKP-25 complexed with the six coronavirus spike targets. Each per-replicate value was averaged over 100 frames from the stable 50–100 ns production window; entries are the mean ± standard deviation across three independent replicates. SARS-CoV-2 (6M0J) is the experimentally validated reference system. The HCoV-229E value averages over distinct surface sites rather than a single binding mode (Section 2.5).

System (PDB)	$\Delta E_{\mathrm{vdw}}$	$\Delta E_{\mathrm{el}}$	$\Delta G_{\mathrm{GB}}$	$\Delta G_{\mathrm{surf}}$	$\Delta G_{\mathrm{bind}}$
MERS-CoV (4KR0)	−38.89±3.57	−3.01±0.87	11.50±1.44	−4.18±0.44	−34.57±3.41
HCoV-NL63 (3KBH)	−35.16±4.39	−4.24±1.53	11.25±2.51	−3.50±0.28	−31.66±3.54
SARS-CoV-2 (6M0J)	−33.11±4.85	−4.11±2.21	11.21±0.12	−3.15±0.70	−29.15±3.46
HCoV-HKU1 (8Y7Y)	−31.74±10.92	−2.74±3.30	9.58±4.64	−3.22±1.09	−28.12±10.61
HCoV-229E (6ATK)	−31.65±7.11	−2.96±1.00	9.67±0.91	−3.06±0.62	−28.00±7.18
SARS-CoV-1 (2AJF)	−28.53±2.09	−2.36±1.20	9.52±1.85	−2.91±0.18	−24.28±1.46

**Table 4:** Target-specific GNINA docking search boxes. Centers and dimensions are given in the coordinate frame of the corresponding PDB entry. Each box encloses the native receptor-recognition hotspot residues plus a 4.0 Å buffer on all six sides.

System (PDB)	Box Center (Å)			Box Size (Å)		
	x	y	z	x	y	z
MERS-CoV (4KR0)	−43.00	25.00	29.00	59.00	35.00	23.00
HCoV-NL63 (3KBH)	7.47	0.60	43.00	17.00	36.00	18.00
SARS-CoV-1 (2AJF)	9.00	−17.00	70.00	17.00	39.00	29.00
HCoV-HKU1 (8Y7Y)	140.00	131.00	148.00	20.00	25.00	81.00
HCoV-229E (6ATK)	115.80	99.00	55.00	23.00	27.00	34.00
SARS-CoV-2 (6M0J)	−35.00	31.00	1.00	20.00	40.00	20.00

## Data Availability

The original data presented in the study are openly available in GitHub at https://github.com/aggelos-michael-papadopoulos/ckp25-broad-spectrum-coronavirus-candidate (accessed on 2 October 2026).

## Supplementary Materials

The following supporting information is available online https://www.mdpi.com/article/10.3390/ph19101664/s1: Table S1: Docking search-box center coordinates and dimensions for CKP-25 focused interface docking; Table S2: Expanded molecular dynamics summary statistics; Table S3: Molecular dynamics preparation and simulation parameters encoded in the GROMACS workflow. Additional machine-readable CSV tables include Table S4: Top 20 residue contact frequencies; Table S5: Per-frame MM-GBSA energy values; Table S6: Per-replicate MM-GBSA energy components; Table S7: Per-replicate random seeds; Table S8: HCoV-229E per-replicate contact sites; and Table S9: SARS-CoV-2 Tyr505 occupancy per replicate.

## Abbreviations

The following abbreviations are used in this manuscript:

HCoV	Human Coronavirus
SARS	Severe Acute Respiratory Syndrome
MERS	Middle East Respiratory Syndrome
RBD	Receptor-Binding Domain
CKP-25	C_19_H_16_N_2_O
MD	Molecular Dynamics
MM-GBSA	Molecular Mechanics–Generalized Born Surface Area
RMSD	Root-Mean-Square Deviation
RMSF	Root-Mean-Square Fluctuation
Rg	Radius of Gyration
SASA	Solvent-Accessible Surface Area
PDB	Protein Data Bank
CNN	Convolutional Neural Network
PME	Particle Mesh Ewald
LINCS	Linear Constraint Solver
GAFF2	General Amber Force Field 2
